# A novel hybrid model integrating MFCC and acoustic parameters for voice disorder detection

**DOI:** 10.1038/s41598-023-49869-6

**Published:** 2023-12-20

**Authors:** Vyom Verma, Anish Benjwal, Amit Chhabra, Sunil K. Singh, Sudhakar Kumar, Brij B. Gupta, Varsha Arya, Kwok Tai Chui

**Affiliations:** 1 Department of Computer Science and Engineering, Chandigarh College of Engineering and Technology, Sector-26, Chandigarh, India; 2 Present Address: Department of Computer Science and Information Engineering, Asia University, Taichung, 413 Taiwan; 3 Kyung Hee University, 26 Kyungheedae-ro, Dongdaemun-gu, 02447 Seoul, Korea; 4 Symbiosis Centre for Information Technology (SCIT), Symbiosis International University, Pune, India; 5 Department of Electrical and Computer Engineering, Lebanese American University, 1102 Beirut, Lebanon; 6 Center for Interdisciplinary Research, University of Petroleum and Energy Studies (UPES), Dehradun, India; 7 Department of Business Administration, Asia University, Taichung, 413 Taiwan; 8 Department of Electronic Engineering and Computer Science, School of Science and Technology, Hong Kong Metropolitan University (HKMU), Kowloon, Hong Kong

**Keywords:** Biological techniques, Health care, Medical research, Mathematics and computing

## Abstract

Voice is an essential component of human communication, serving as a fundamental medium for expressing thoughts, emotions, and ideas. Disruptions in vocal fold vibratory patterns can lead to voice disorders, which can have a profound impact on interpersonal interactions. Early detection of voice disorders is crucial for improving voice health and quality of life. This research proposes a novel methodology called VDDMFS [voice disorder detection using MFCC (Mel-frequency cepstral coefficients), fundamental frequency and spectral centroid] which combines an artificial neural network (ANN) trained on acoustic attributes and a long short-term memory (LSTM) model trained on MFCC attributes. Subsequently, the probabilities generated by both the ANN and LSTM models are stacked and used as input for XGBoost, which detects whether a voice is disordered or not, resulting in more accurate voice disorder detection. This approach achieved promising results, with an accuracy of 95.67%, sensitivity of 95.36%, specificity of 96.49% and f1 score of 96.9%, outperforming existing techniques.

## Introduction

In a number of social circumstances, changes in voice tone reveal a great deal about the speaker. Voices provide information about a person’s gender, age, geographical and socioeconomic background, education, and employment. Every voice is different in its authenticity and originality. A variety of things impact the efficacy and quality of a voice. Globally, the number of people experiencing voice-related impairments is on a concerning rise. For example, in the United States alone, approximately 18 million adults report issues with their voices annually^1,2^, with approximately 10 million individuals suffering from persistent voice problems^3^. Children are not exempt from these challenges either, as the National Institute on Deafness and Other Communication Disorders (NIDCD) indicates that one in every 12 children grapples with disorders linked to voice, speech, language, or swallowing^4^.

To produce voice, the vocal folds and vocal tract regulate and resonate air pressure vibrations expelled from the lungs. The respiratory system controls the pressure behind the vocal folds during expiration, which affects voice volume. The larynx plays a crucial role in voice production, primarily through the vibration of the vocal folds, which determines the quality of sound. Additionally, the vocal tract, comprising the pharynx and associated nasal and paranasal structures, also contributes significantly^5,6^. There is also the central nervous system and the auditory system. Some systems involved in voice production, such as breathing and pneumophonic coordination, are governed by the latter, and several of them are managed by the central nervous system. Certain diseases can lead to an alteration in this mechanism, either functionally or morphologically, resulting in degraded voice quality and intensity. Dysphonia is the medical term for voice disorders. There are different types of voice abnormalities, including nodules, cysts, and paralysis^7,8^. Vocal fold abnormalities interrupt voice patterns, causing a disordered individual’s speech signal to be more transitory and loud than that of a healthy individual^9^. Globally, there are several types of voice disorders that affect millions of people. There are a number of risk factors associated with the development of these disorders, including vocal abuse and incorrect lifestyle habits, including smoking and alcohol consumption. Voice disorders must be evaluated in terms of their characteristics and their health effects.

Voice disorders, encompassing a range of conditions from benign vocal fold nodules to more severe pathologies, have gained prominence, particularly in the era of COVID-19, with altered communication patterns and increased mask usage^10^. Early and accurate detection of these disorders is essential for improving the quality of life of affected individuals. Voice pathologies can be caused on the surface of the vocal folds due to factors such as not drinking enough water, smoking, drinking too much alcohol, or using your voice too much^11^. Because of these diseases, the vocal folds do not open and close at regular times and vibrate in a strange way. When this happens, the vocal folds make sounds that are less clear, harsher, and strained. Because of such problems with the vocal folds, these voices send out considerable noise. This makes it hard for the human ear to listen to these voices. There are many voice disorders that can be caused by smoking cigarettes. As a result of chronic use, the larynx can become inflamed, erythematous, dry, and itchy. Laryngeal reflux, Reinke’s edema, and laryngeal carcinoma are also caused by smoking. Laryngeal disease is often characterized by a disordered voice as the first symptom, often due to pathological changes in the larynx. The effects of smoking on perception, acoustics, and aerodynamics during phonation have also been studied previously^12^.

The ability to detect and diagnose voice disorders accurately is crucial for early intervention and effective treatment. In this paper, a novel hybrid model that combines artificial intelligence and machine learning (AI/ML) techniques^13^ that integrates mel-frequency cepstral coefficients (MFCCs) for voice disorder detection is presented. MFCC, a powerful tool for acoustic feature extraction^14^, is integrated into the hybrid model to enhance the precision and reliability of voice disorder detection. The objectives of the paper are as follows:

1. To study the background and provide a literature review with regard to voice disorder detection using machine learning algorithms.
2. To propose a novel methodology, i.e., VDDMFS (voice disorder detection using MFCC, fundamental frequency and spectral centroid) to solve the problem of voice disorder detection. The novelty of the VDDMFS lies in its integration of multiple acoustic features to enhance accuracy and reliability in voice disorder detection. It makes use of LSTM, ANN and Xgboost algorithms to create a hybrid model to detect disorder in voice.
3. To test and validate the proposed methodology on diverse performance metrics such as accuracy, specificity and sensitivity.
4. To compare the proposed methodology with existing techniques such as OSELM (online sequential extreme learning machine)^15^, DDI (Dysphonia Detection Index)^16^ and other techniques using CNN, SVM, MLP and filter banks.

The remaining sections are organized as follows. In “Literature review” of this paper, the literature review is discussed. “Materials and methods” covers the methodology, including the dataset used, data preprocessing and feature extraction steps, and the models used. The proposed methodology is discussed in “Methods”. “Experimentation, results and analysis” discusses the experimentation and results. Finally, conclusions and future scope are provided in “Results”.

## Literature review

This literature review aims to explore and critically examine the existing research efforts, methodologies, and advancements in the application of machine learning and deep learning techniques for voice disorder detection. From emotion recognition^14,17^ to state recognition in healthcare^13,18,19^, speech and voice signals are used in many applications. The state of voice health is estimated by many m-health systems using these signals^20,21^. Many approaches to improving the accuracy of detecting pathology in a voice have been developed recently, mainly using machine learning techniques^22,23^. The studies are aimed at identifying parameters of voice quality and identifying methods of detecting disorders of the voice. Machine learning algorithms and specially developed acoustic characteristics were key to many of the methods presented in the literature.

Grzywalski et al.^24^ introduced a deep neural network (DNN)-based system for the detection of multiple voice disorders. The system achieved an accuracy of 77.4, which is the weighted average of sensitivity and specificity. A final sensitivity of 92.0% and specificity of 85.9% were achieved.

Verde et al.^25^ used the SVD dataset^26^ along with support vector machine and decision tree algorithms to attain an accuracy of approximately 86%. Harar et al.^27^ used XGBoost, which demonstrated the best classification performance with an F1 score of 0.733 when using acoustic (dysphonic) features (AF) and mel-frequency cepstral coefficients (MFCC). Verde et al.^16^ introduced a novel marker known as the “dysphonia detection index” to assess voice health and identify voice disorders. The proposed dysphonia detection index demonstrated its effectiveness, achieving an accuracy of 82.2%, with sensitivity and specificity reaching 82% and 82.6%, respectively.

Tulics et al.^28^ compared two types of input vectors: acoustic parameters and phone-level posterior probabilities acquired from a deep neural network (DNN) soft-max layer in a speech recognition system. They attained an accuracy of 85% using DNN. AL-Dhief et al.^15^ introduced a voice pathology detection system based on an online sequential extreme learning machine (OSELM) for classifying voice signals as healthy or pathological, with feature extraction using mel-frequency cepstral coefficients (MFCCs). The results demonstrated a maximum accuracy, sensitivity, and specificity of 85%, 87%, and 87%, respectively, leaving room for further improvement.

Chui et al.^29^ introduced the CGAN-IFCM model, which combines a conditional generative adversarial network (CGAN) with improved fuzzy C-means (IFCM) clustering for multiclass voice disorder detection. Their approach improved sensitivity (9.9–12.9%) and specificity (9.1–44.8%) marginally, but no improvement in accuracy of the model was seen for binary classification.

Abakarim and Abenaou^30^ employed the adaptive orthogonal transform method for feature extraction. Support vector machine (SVM) and multilayer perceptron (MLP) models were utilized, achieving an accuracy of 85.79%. Sztaho et al.^31^ introduced a deep learning approach aimed at detecting pathological voice disorders within continuous speech. They proposed a solution that combines a long short-term memory (LSTM) autoencoder with multitask learning, using spectrograms as input features. It achieved an accuracy of 86% for disorder detection.

Islam et al.^32^ introduced a voice pathology detection approach employing convolutional neural networks (CNNs) with electroglottographic (EGG) and speech signals. Their two-step CNN system achieved an accuracy of 80.30% for binary classification using raw temporal speech signals. However, the study relied on the Saarbruecken Voice Database (SVD), an outdated dataset that warrants consideration. The accuracy is also not good enough to be used in a robust system.

Islam et al.^33^ examined two cochlear implant models in their study. The research findings revealed that both proposed models utilizing bandpass and gammatone filter banks yielded F1 scores of 77.6% and 78.7%, respectively, when analyzing speech samples. However, it is noteworthy that the first model achieved validation and testing accuracies of 85.96% and 77.91%, respectively, while the second model achieved accuracies of 81.98% and 77.5%, respectively, indicating that these methods may not represent the optimal choice for pathological voice identification.

**Figure 1:**
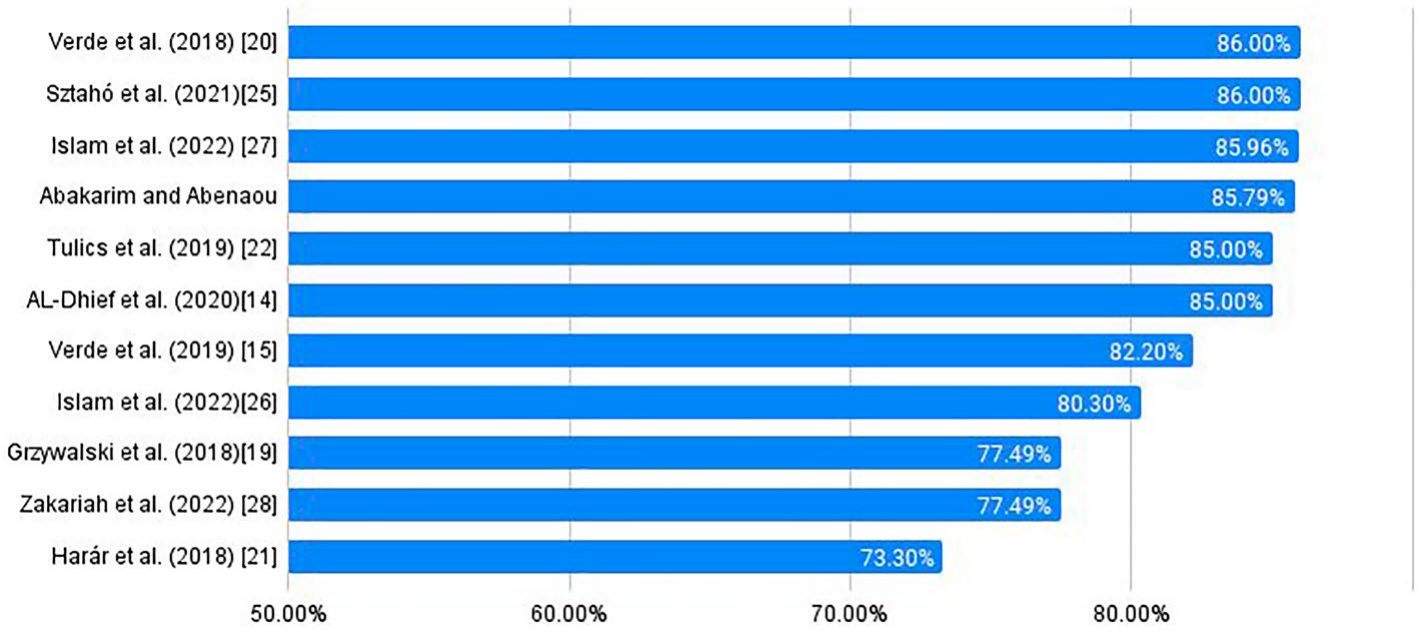
Accuracy comparisons of previous studies.

Zakariah et al.^34^ presented a framework that integrated three essential voice characteristics: chroma, mel spectrogram, and mel frequency cepstral coefficient (MFCC). They used a deep neural network (DNN) to attain an accuracy of 77.49%. The findings can be summarized as shown in Table 1 and visualized in Fig. 1.

**Table 1:** Summary of studies examining the detection of voice disorders.

Source	Dataset used	Method	Results
Grzywalski et al. (2018)^24^	FEMH voice data challenge	Deep neural network (DNN)	Support vector accuracy: 77.49%
Verde et al. (2018)^25^	SVD	Support vector machine and decision tree algorithm	Accuracy: 86%
Harár et al. (2018)^27^	MEEI + SVD + AVDP	XgBoost algorithm	Accuracy: 73.3%
Verde et al. (2019)^16^	VOICED + MEEI + SVD	DDI (dysphonia detection index)	Accuracy: 82.2%
Tulics et al. (2019)^28^	Voice disordered and healthy adults speech database	Deep Neural Network (DNN)	Accuracy: 85%
AL-Dhief et al. (2020)^15^	SVD	Online sequential extreme learning machine (OSELM)	Accuracy: 85%
Chui et al. (2020)^29^	SVD + VOICED	Conditional generative adversarial network (CGAN)	Sensitivity (9.9–12.9%) and specificity(9.1–44.8%)
Abakarim and Abenaou (2021)^30^	SVD	SVM and multilayer perceptron (MLP)	Accuracy: 85.79%
Sztahó et al. (2021)^31^	Voice Disorder Speech Dataset (VDSD)	Long short-term memory (LSTM)	Accuracy: 86%
Islam et al (2022)^32^	SVD	Convolutional neural networks (CNNs) with electroglottography (EGG)	Accuracy: 80.30%
Islam et al. (2022)^33^	SVD	Bandpass and gammatone filter banks	Accuracy: 85.96%
Zakariah et al. (2022)^34^	SVD	Deep neural network (DNN)	Accuracy: 77.49%

The mentioned studies have notable limitations that warrant attention^35^. Firstly, they often concentrate only on specific features like MFCC or fundamental frequency in voice disorder detection, potentially restricting the models' ability to fully comprehend voice signals. Secondly, reliance on outdated datasets introduces biases and compromises the relevance of findings in the rapidly evolving landscape of voice disorder detection. Lastly, a common limitation is the use of a narrow set of evaluation metrics, which might offer only a partial understanding of model performance. This research aims to overcome these issues by adopting a broader approach, incorporating diverse features such as MFCC, spectral centroid, fundamental frequency, age, and gender. It utilizes up-to-date datasets and employs a wider range of evaluation metrics, including sensitivity, specificity, and F1 score. These enhancements ensure a more comprehensive and nuanced perspective on voice disorder detection.

## Materials and methods

The subsequent sections introduce the dataset used in this study, the preprocessing performed and the machine learning techniques used.

### Materials

#### Dataset description

In this research work, the VOICED^36^ (VOice ICar fEDerico II) dataset was used to analyze the different voice disorders. A total of 208 clinically verified voice samples are included in this dataset, 150 of which are pathological and 58 of which are healthy. Participants in the study needed to be between 18 and 70 years old and capable of adhering to the anticipated ages of the research procedure^28,37,38^. VOICED used a built-in microphone of a mobile device^39^ and an applicable m-health system, Vox4Health^40,41^, to collect a speech signal of vowel ‘a’ in real time. In every recording, the sample rate was set at 8000 Hz, and the resolution was set at 32 bits. A filter was also applied to each recording during acquisition to remove any noise that might have been accidentally introduced^42^. The participants were instructed to maintain constant levels of voice intensity while articulating the vocal sample, as if they were conversing normally. The types of dysphonia mentioned in the dataset are as follows:

1. Hyperkinetic dysphonia: hyperkinetic dysphonia is a type of voice disorder characterized by excessive muscle tension and movements in the larynx, which can lead to hoarseness, strain, and other vocal problems.
2. Hypokinetic dysphonia: hypokinetic dysphonia refers to a type of speech disorder that is caused by reduced movement of the vocal cords due to Parkinson’s disease (a neurological condition).
3. Reflux laryngitis: reflux laryngitis is a condition where stomach acid flows back into the throat, irritating the sensitive tissues of the larynx^43^. This can lead to symptoms such as hoarseness, chronic cough, and throat discomfort. It is often caused by gastroesophageal reflux disease (GERD) and requires medical management to alleviate symptoms and prevent complications.

**Figure 2:**
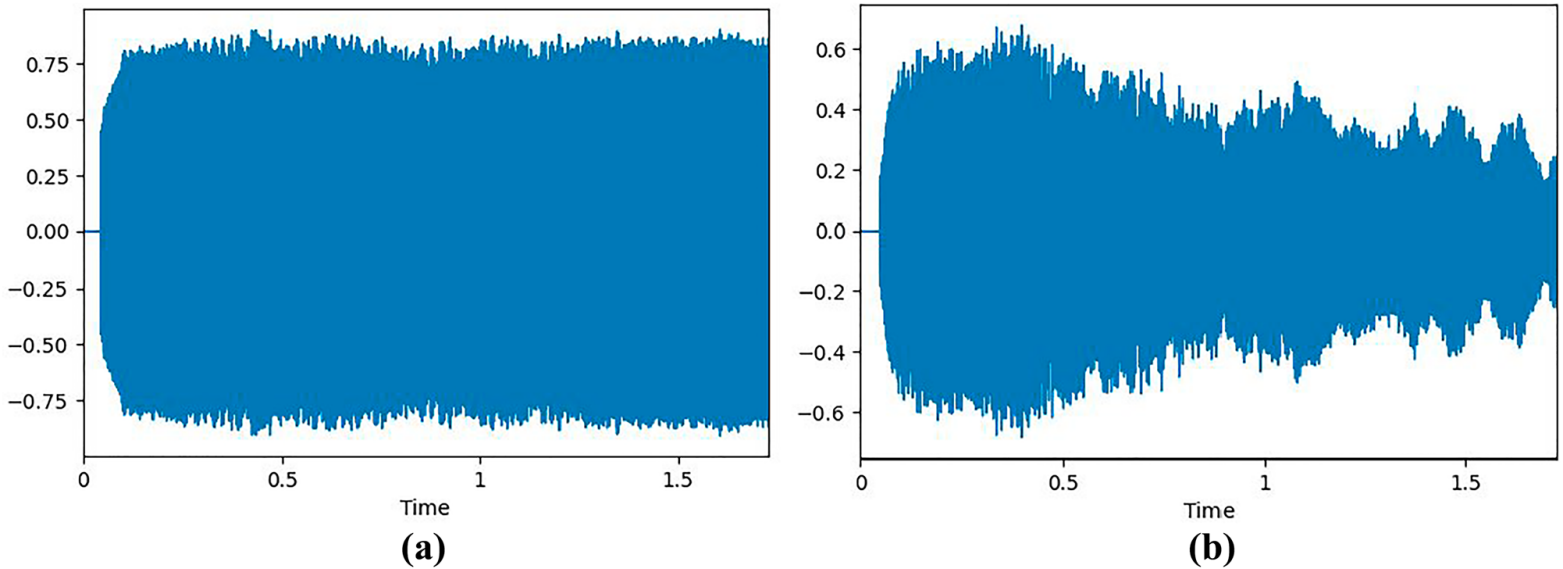
(**a**) Sample wave plot of an unhealthy voice. (**b**) Sample wave plot of a healthy voice.

A summarization of the dataset is shown in Table 2, in which the healthy and pathological voices of both genders and the percentage with respect to the whole dataset are shown. A sample plot of unhealthy and healthy voices is depicted in Fig. 2.

**Table 2:** Summary of the VOICED Dataset with respect to voice disorder.

Dataset	Category	Gender	Count	Percentage (%)
Voiced	Healthy	Female	37	17.78
		Male	21	10.09
	Pathological	Female	98	47.11
		Male	52	25
Total	Healthy	All	58	27.88
	Pathological	All	150	72.11

#### Data preprocessing

**Figure 3:**
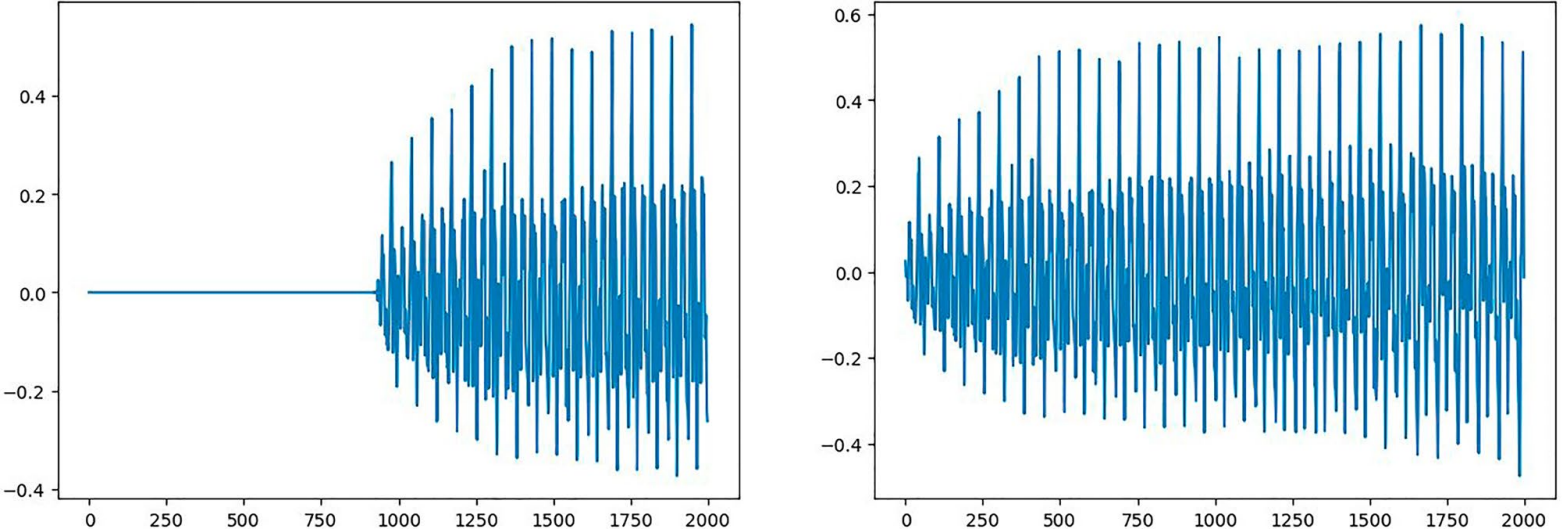
Voice sample signal of a person after trimming silence.

In this research work, the data preprocessing approach plays a pivotal role in enhancing the quality and relevance of the data used for the study. The first step involves the extraction of essential features from voice samples. Voice data often contain periods of silence, which can introduce noise and inefficiencies into subsequent analyses. To address this issue, the voice samples were initially trimmed, effectively removing silence and ensuring that the extracted features were derived from meaningful vocalizations. After initial trimming of silence, the voice sample can be visualized as shown in Fig. 3.

The core of the feature extraction process centers around the extraction of 20 Mel-frequency cepstral coefficients (MFCCs), a widely used representation for characterizing the spectral content of voice signals^44,45^. MFCCs offer valuable insights into the acoustic properties of voice samples, allowing for the capture of critical information pertaining to speech patterns and vocal characteristics. Additionally, feature extraction is extended to encompass the fundamental frequency (f0)^46^ and spectral centroid, which further contribute to understanding voice data^47–49^. Demographic information, specifically age and sex, was also incorporated into the analysis. Recognizing the importance of these factors in voice-related research, age and sex data were included with the fundamental frequency and spectral centroid features^43,50–53^. This holistic approach enables the exploration of potential correlations and interactions between these demographic variables and the acoustic properties of the voice, contributing to a more comprehensive understanding of the dataset. Consider a small subset of the dataset containing five rows of voice samples shown in Table 3.

**Table 3:** A small subset of extracted data.

id	f0	Spectral centroid	Age	Sex	Diagnoses
voice001	112.919473	1185.915658	32	M	Disordered
voice002	128.547035	1019.691265	55	M	Healthy
voice003	143.810626	1183.157022	34	M	Disordered
voice004	191.597747	1318.389801	28	F	Disordered
voice005	163.848356	1400.755172	54	F	Disordered

Subsequently, in the preprocessing phase dedicated to MFCCs, the issue of variable sequence lengths was addressed. The length of voice samples can vary significantly, which can complicate subsequent modeling and analysis. To ensure uniformity, the MFCC sequences were padded, bringing them all to the same shape. This preprocessing step ensures that data are amenable to a wide range of machine learning and statistical techniques, as it eliminates discrepancies in sequence lengths. Moreover, recognizing the importance of data scaling in the modeling process, a standard scaler was applied to the MFCC data. This scaling technique standardizes the feature values, placing them on a common scale, which is particularly crucial when employing algorithms that are sensitive to the magnitude of input features^54^. This preprocessing step aids in the stability and convergence of subsequent modeling efforts, ensuring that all features contribute equally to the analysis. For the fundamental frequency, spectral centroid, age, and sex features, first, label encoding on the sex variable was performed, converting it into a numeric format suitable for analysis. Following encoding, standard scaling was applied to the entire set of features. By standardizing the data in this manner, all variables are treated consistently during modeling, reducing the potential for biases stemming from differences in feature scales. In summary, the data preprocessing approach encompasses the removal of silence from voice samples, feature extraction of MFCCs, fundamental frequency, and spectral centroid, as well as the incorporation of age and sex data. To facilitate uniformity, MFCC sequences were padded, and then standard scaling was applied to both the MFCCs and demographic features. This meticulous preprocessing pipeline lays the foundation for robust and insightful analyses, enhancing the quality and reliability of the results obtained in this research.

## Methods

In this section, the methodologies employed in this research are outlined to address the task of voice disorder classification. The utilization of an artificial neural network (ANN), a long short-term memory (LSTM), and an XGBoost model is presented, each contributing uniquely to enhancing the accuracy and robustness of the classification framework.

- Long short-term memory (LSTM): LSTM, a type of recurrent neural network (RNN), excels in sequence-based classification tasks by retaining and utilizing information from past inputs^55^. Its specialized architecture with memory cells allows it to capture intricate dependencies within sequential data, making it a valuable choice for applications such as time series prediction and natural language processing.
- Artificial neural network (ANN): ANNs are versatile tools for classification tasks that are capable of processing diverse data types^56–58^. These networks consist of layers of interconnected nodes that adapt and learn from labeled training data^28^. ANNs are known for their capacity to uncover intricate patterns and relationships within data, making them applicable to a wide range of classification problems^59–61^
- XGBoost (extreme gradient boosting): XGBoost is a potent ensemble learning algorithm for classification tasks. It operates by iteratively combining the predictions of multiple weak models, enhancing accuracy through a focus on misclassified data points^27^. XGBoost is recognized for its speed and ability to handle complex, structured data, making it a popular choice for achieving high predictive performance in various machine learning applications.

### Proposed methodology

**Figure 4:**
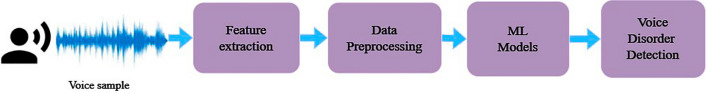
Flowchart of a general process for voice disorder detection.

In this section, a methodology called voice disorder detection using MFCC, fundamental frequency and spectral centroid (VDDMFS) is proposed. A general approach is shown in Fig. 4. This method combines MFCC with fundamental frequency and spectral centroid to predict whether a voice is pathological. In the proposed methodology for voice disorder detection, a comprehensive set of acoustic features, including Mel-frequency cepstral coefficients (MFCCs), spectral centroid, and fundamental frequency (F0) were integrated to facilitate the accurate detection of voice disorders.

Leveraging the power of deep learning, this approach incorporates both long short-term memory (LSTM) networks for MFCC and artificial neural networks (ANNs) for other parameters to capture intricate patterns in voice samples. Furthermore, to optimize predictive performance and enhance model robustness, the ensemble learning technique with XGBoost as a meta-model was employed. This integrated framework, acronymically represented as VDDMFS, aims to provide an effective and efficient solution for the detection of voice disorders through the fusion of multiple modalities and advanced machine learning methodologies.

### System model

VDDMFS is designed for the task of detecting voice disorders by fusing a number of acoustic features. The features encompass Mel-frequency cepstral coefficients (MFCCs), spectral centroid, and fundamental frequency (F0). The mathematical model for the same can be described as follows:

- Feature extraction
- MFCC extraction: let XMFCC be the matrix of MFCC features for voice samples, where each row xMFCC represents the MFCC coefficients for a voice sample.
- Age, sex, F0, and spectral centroid extraction: let XMetadata be the matrix of metadata features, including age, sex, F0, and spectral centroid, where each row xMetadata represents the metadata features for a voice sample.
- Model training
- LSTM model for MFCC: the LSTM model processes the MFCC features: HMFCC = LSTM(XMFCC). HMFCC represents the hidden states of the LSTM for the MFCC features.
- ANN model for age, sex, F0, and spectral centroid: the ANN model processes the metadata features: YMeta- data = ANN(XMetadata) YMetadata represents the output of the ANN for the metadata features.
- ***Probability estimation***
- Probabilities from LSTM and ANN: the LSTM and ANN models provide probability vectors for each voice sample: PLSTM for LSTM PANN for ANN.
- ***Ensemble with XGBoost***
- Stacking probabilities: probabilities from both models have been stacked into a feature matrix XStacked = [PLSTM,PANN]
- XStacked is used as input to the XGBoost model.
- ***Final classification***
- XGBoost classification: the XGBoost model classifies voice samples: YXGBoost = XGBoost (XStacked)
- YXGBoost represents the final classification output.
- ***Evaluation***
- Performance metrics: the system’s performance was evaluated using standard binary classification metrics, including accuracy, precision, and recall.

### Architecture and working

**Figure 5:**
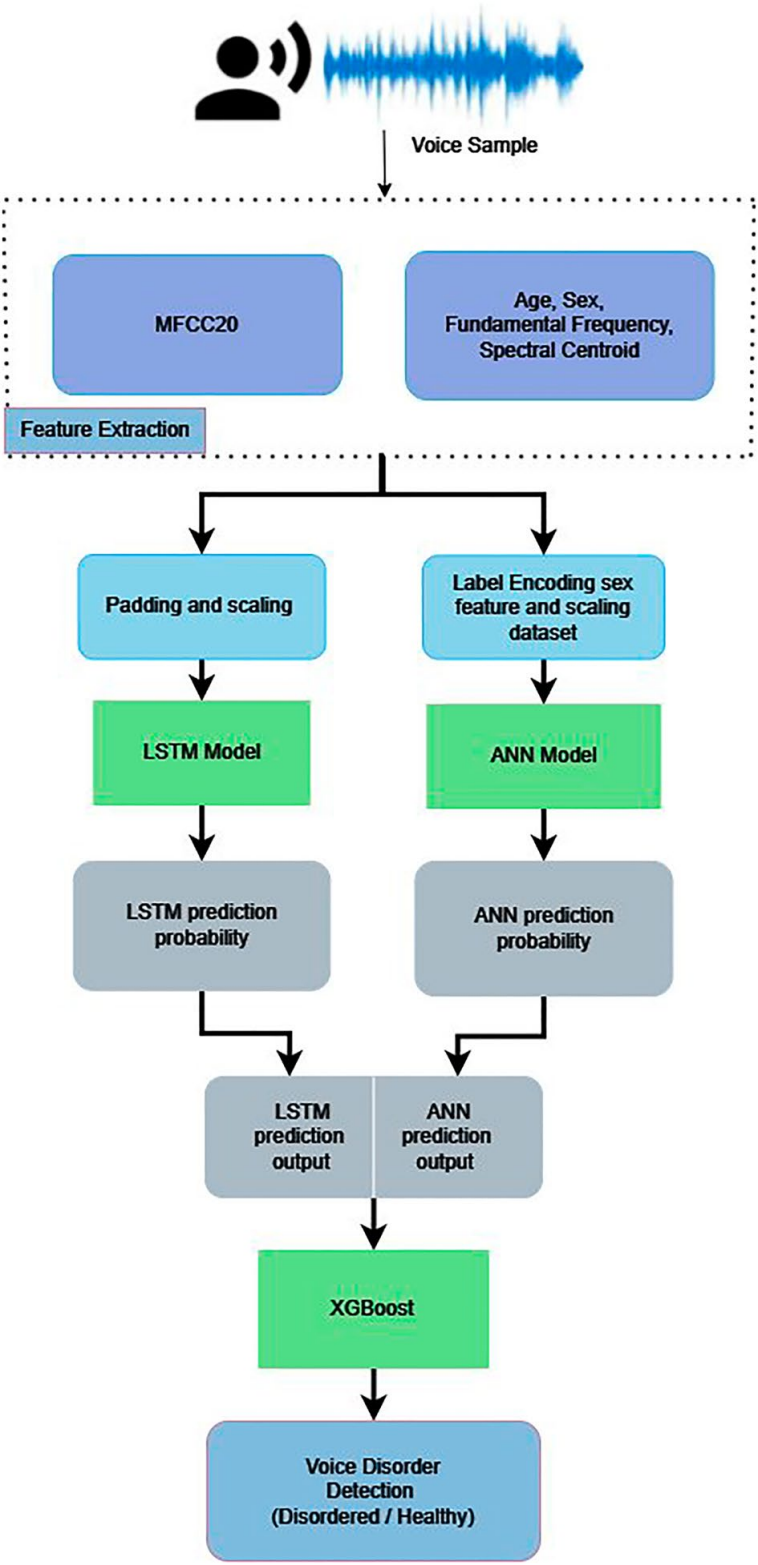
Flowchart illustrating the proposed methodology.

In voice disorder detection using MFCC, fundamental frequency and spectral centroid (VDDMFS), the algorithm was initiated by extracting the main features from the voice sample. Mel-frequency cepstral coefficients (MFCCs) are extracted from the input voice signal, alongside critical metadata features such as the fundamental frequency (f0), spectral centroid, age, and sex, which provide comprehensive insights into the voice sample. For model training and label encoding, the algorithm converted the “sex” metadata feature into a binary format using a label encoder. It subsequently applied standard scaling to normalize the f0, spectral centroid, age, and sex features, ensuring uniformity in their scales. The LSTM Model was employed to predict voice disorder probability from the sequential MFCC data, leveraging its capability to capture temporal dependencies. Concurrently, the ANN Model processed the nonsequential features f0, spectral centroid, age, and sex to generate another probability value for voice disorder. The two probability values obtained from the LSTM and ANN models were stacked into a feature matrix (X) encapsulating both sequential and nonsequential information. This matrix was used as input into the XGBoost Model, an ensemble learning algorithm, producing the final binary classification result for voice disorder. The ultimate output of the algorithm, denoted as prediction, conveyed the presence or absence of a voice disorder, with a value of 0 indicating no voice disorder and a value of 1 signifying the detection of a voice disorder. The flowchart of the algorithm is depicted in Fig. 5. From the steps mentioned in the flowchart of the proposed approach, the VDDMFS algorithm can be defined as follows.

**Algorithm 1:**
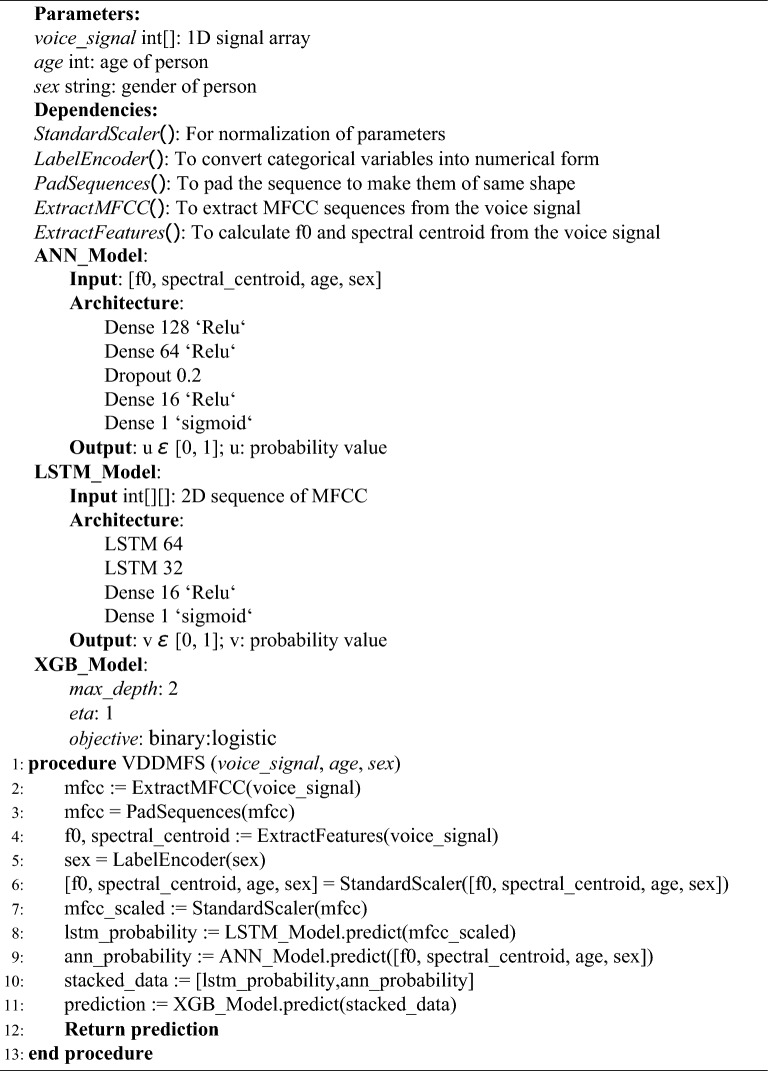
VDDMFS: Voice Disorder Detection using MFCC, Fundamental Frequency and Spectral Centroid.

## Experimentation, results and analysis

In the experimentation, results, and analysis section of this research work, the focus shifts to a comprehensive examination of the empirical investigation conducted to evaluate the proposed voice disorder detection using MFCC, fundamental frequency and spectral centroid (VDDMFS). This section serves as the empirical cornerstone of the study, presenting the outcomes of meticulously designed experiments and providing insightful analyses of the findings. Through a systematic exploration of the model’s performance, the effectiveness in accurately detecting voice disorders is assessed.

### Experimental setup

The research work was conducted on a system equipped with the following hardware specifications:

- CPU: an Intel Skylake CPU with 4 cores was used for computational tasks. This CPU configuration provided sufficient processing power for the deep learning model^62,63^.
- RAM: the system had 30 GB of RAM, which allowed for efficient data handling and model training^64^.
- Disk space: a total of 20 gigabytes of disk space were available for storing datasets, code files, and research artifacts. This disk space was crucial for data storage and management throughout the research process.

The primary programming language employed for the research work was Python. Python’s versatility and extensive ecosystem of libraries make it a suitable choice for data preprocessing, model development, and analysis. Several Python libraries, including TensorFlow, NumPy, scikit-learn, XGBoost, Librosa^65^, Pandas, Matplotlib, WFDB^49,66^ and Seaborn, were utilized for various tasks.

### Performance metrics

The evaluation of the system's effectiveness in distinguishing between normal and pathological voices encompassed a comprehensive set of performance metrics. Accuracy, sensitivity, specificity and F1 score were utilized to provide a more nuanced understanding of VDDMFS’s performance.

The accuracy represents the overall correctness of the model in classifying both normal and pathological voices. It is calculated as follows:

$$\mathrm{Accuracy }(\mathrm{\%}) = (\mathrm{TP }+\mathrm{ TN}) / (\mathrm{TP }+\mathrm{ FN }+\mathrm{ FP }+\mathrm{ TN})$$

The sensitivity measures how effectively the model classifies pathological voices, capturing the true positive rate. The sensitivity (percentage) is calculated as:

$$\mathrm{Sensitivity }(\mathrm{\%}) =\mathrm{ TP }/ (\mathrm{FN }+\mathrm{ TP})$$

Specificity indicates the model's ability to correctly identify healthy voices, representing the true negative rate. It is calculated as:

$$\mathrm{Specificity }(\mathrm{\%}) =\mathrm{ TN }/ (\mathrm{FP }+\mathrm{ TN})$$

F1 Score provides a balance between precision and recall, offering insight into the model's ability to minimize false positives and false negatives simultaneously. It is calculated as:

$${\text{F}}1\,\mathrm{ Score }(\mathrm{\%}) = 2 * (\mathrm{Precision }*\mathrm{ Recall}) / (\mathrm{Precision }+\mathrm{ Recall})$$

Where Recall (%) = TP/(TP + FN) and Precision (%) = TP/(TP + FP).

Parameters defined within these metrics include:

- True positive (TP): the true positive (TP) marker recognizes pathology in voice samples.
- True negative (TN): a true negative (TN) is shown because the marker can tell that the voice sample is from a perfectly healthy person.
- False positive (FP): when the marker identifies a healthy speech sample as having pathological characteristics despite the voice sample being healthy.
- False negative (FN): although the voice sample was taken from a patient with a pathological condition, the marker deemed it to be healthy.

## Results

In this subsection, training loss and accuracy graphs for both the LSTM and ANN models are presented to offer deeper insights into the training dynamics and convergence of the proposed voice disorder detection using MFCC, fundamental frequency, and spectral centroid (VDDMFS). Subsequently, the effectiveness of the VDDMFS in accurately detecting voice disorders is demonstrated through rigorous evaluation utilizing key performance metrics, including accuracy, sensitivity, and specificity.

### Training of LSTM and ANN

In this subsection, the training process of the LSTM and ANN models is explained, peering into the intricacies of their learning dynamics. Through the presentation of training loss and accuracy curves, how these models evolve and converge during the training phase can be elucidated.

#### LSTM model

The LSTM model was trained for 25 epochs with the Adam optimizer and binary cross entropy as the loss function.

**Figure 6:**
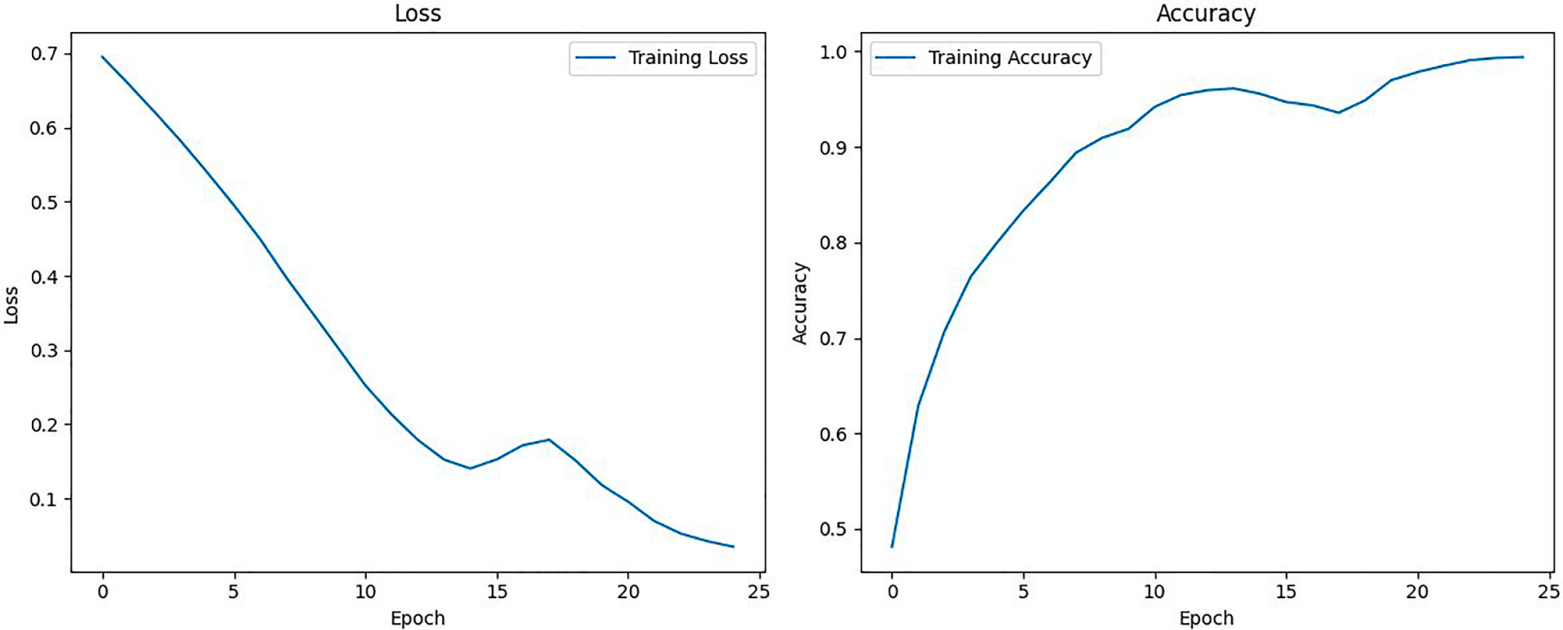
Training loss and accuracy curves for the LSTM model.

In the training phase of the LSTM model, which was designed to learn from the extracted MFCC features, noteworthy observations emerged from the training curve. As shown in Fig. 6, initially, during the early epochs, both training loss and accuracy exhibited substantial fluctuations as the model began to grasp intricate patterns from the MFCC data. As training progressed, these fluctuations gradually diminished, leading to a steady decrease in loss, reaching its lowest point at the 14th epoch. Following this phase of steady decline, there was a brief increase in the loss, a phenomenon often referred to as a ’loss spike,’ likely triggered by subtle variations or noise in the training data. Importantly, after the 17th epoch, the training loss continued to decrease, indicating the model’s resilience in overcoming the transient challenge and improving its understanding of the MFCC voice features. The convergence of the training loss and accuracy curves underscored the LSTM model’s adaptability and its capacity to learn complex voice disorder detection patterns from the MFCC data.

### ANN model

In the training phase of the ANN model, which was employed to learn from the extracted features comprising f0 (fundamental frequency), spectral centroid, age, and sex information, distinctive observations emerged from the training curve.

**Figure 7:**
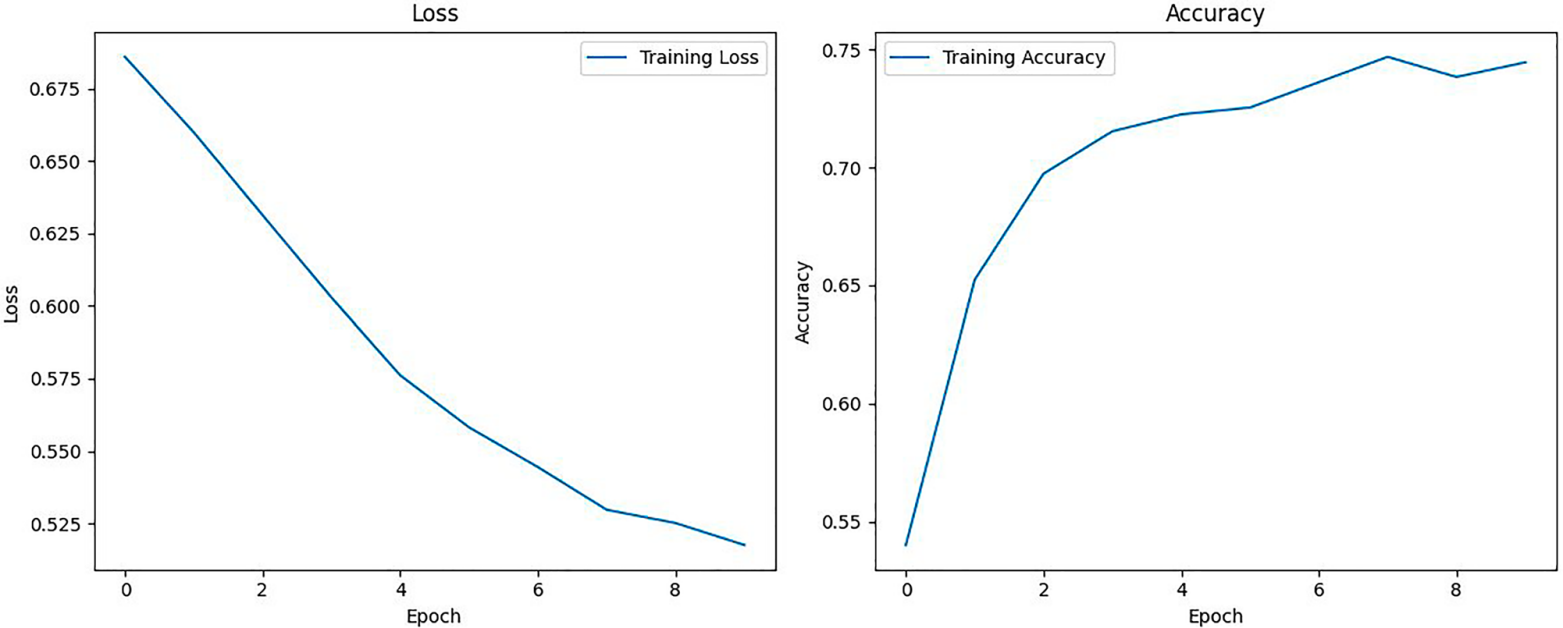
Training loss and accuracy curves for the ANN model.

Unlike the LSTM model, the ANN model displayed minimal fluctuations in training loss throughout the epochs, demonstrating a consistent and gradual decrease as shown in Fig. 7. This stability in loss signifies the model’s steady learning process, as it assimilated complex patterns from the extracted features. However, a slight variation was observed in the accuracy curve, with momentary fluctuations in accuracy scores. These fluctuations in accuracy, while minor, may reflect the model’s response to subtle variations or nuances in the training data. Importantly, the ANN model’s ability to maintain a consistent decrease in loss demonstrates its resilience and adaptability, ultimately contributing to its understanding of the intricate voice disorder detection patterns encoded in the features. The convergence of the training loss and accuracy curves underscores the ANN model’s capacity to effectively learn from f0, spectral centroid, age, and sex features.

### Performance evaluation of VDDMFS

**Figure 8:**
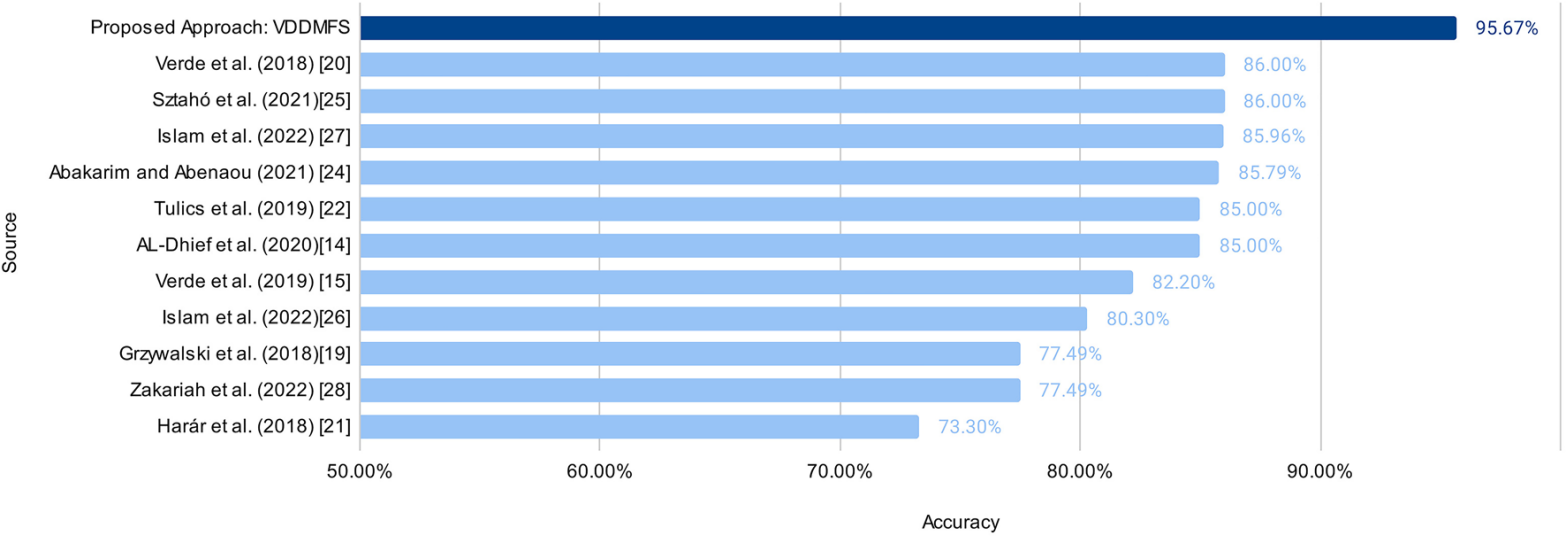
Accuracy comparisons visualizing Table 4.

This section presents a thorough evaluation of voice disorder detection using MFCC, fundamental frequency, and spectral centroid (VDDMFS). Critical metrics, including the F1 score, specificity, sensitivity, and accuracy, are analyzed here to assess the effectiveness of the VDDMFS in voice disorder detection. Comparisons with existing techniques are highlighted in Table 4 and visualized in Fig. 8, showing its potential clinical significance.

**Table 4:** Comparison of the results of the proposed approach with previous studies.

Source	Method	Results
Proposed Approach: VDDMFS	LSTM, ANN stacked using XGBoost	Accuracy: 95.67%
Grzywalski et al. (2018)^24^	Deep neural network (DNN)	Support vector accuracy: 77.49%
Verde et al. (2018)^25^	Support vector machine and decision tree algorithm	Accuracy: 86%
Harár et al. (2018)^27^	XgBoost algorithm	Accuracy: 73.3%
Verde et al. (2019)^16^	DDI (dysphonia detection index)	Accuracy: 82.2%
Tulics et al. (2019)^28^	Deep neural network (DNN)	Accuracy: 85%
AL-Dhief et al. (2020)^15^	Online sequential extreme learning machine (OSELM)	Accuracy: 85%
Chui et al. (2020)^29^	Conditional generative adversarial network (CGAN)	Sensitivity (9.9–12.9%) and specificity (9.1–44.8%)
Abakarim and Abenaou (2021)^30^	SVM and multilayer perceptron (MLP)	Accuracy: 85.79%
Sztahó et al. (2021)^31^	Long short-term memory (LSTM)	Accuracy: 86%
Islam et al (2022)^32^	Convolutional neural networks (CNNs) with electroglottography (EGG)	Accuracy: 80.30%
Islam et al. (2022)^33^	Bandpass and gammatone filter banks	Accuracy: 85.96%
Zakariah et al. (2022)^34^	Deep neural network (DNN)	Accuracy: 77.49%

**Figure 9:**
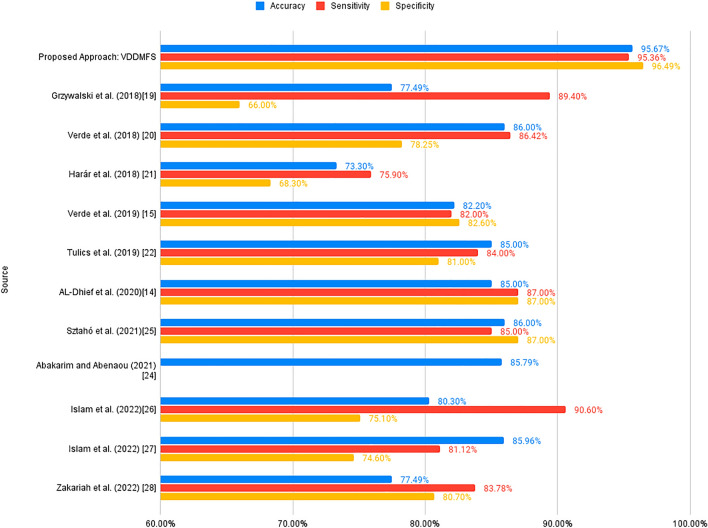
Performance metrics comparison of VDDMFS with previous studies’ results as shown in Table 5.

The results of the study underline the remarkable performance of voice disorder detection using MFCC, fundamental frequency and spectral centroid (VDDMFS) in the context of voice disorder detection. Notably, VDDMFS exhibits a striking accuracy of 95.67%, surpassing the performance of numerous related works and existing techniques in the field. The accuracy, sensitivity and specificity of the proposed model can be compared with other models and methods, as shown in Table 5 and visualized in Fig. 9.

**Table 5:** Classification algorithms and methods comparison for voice disorder detection on the VOICED Dataset.

Source	Results (%)	Sensitivity (%)	Specificity (%)
Proposed approach: VDDMFS	95.67	95.36	96.49
Verde et al. (2018)^25^	86.00	86.42	78.25
Sztahó et al. (2021)^31^	86.00	85	87
Islam et al. (2022)^33^	85.96	81.12	74.60
Abakarim and Abenaou (2021)^30^	85.79	N/S	N/S
Tulics et al. (2019)^28^	85.00	84	81
AL-Dhief et al. (2020)^15^	85.00	87	87
Verde et al. (2019)^16^	82.20	82.00	82.60
Islam et al. (2022)^32^	80.30	90.60	75.10
Grzywalski et al. (2018)^24^	77.49	89.40	66
Zakariah et al. (2022)^34^	77.49	83.78	80.70
Harár et al. (2018)^27^	73.30	75.90	68.30

When juxtaposed with other machine learning models for voice disorder detection, voice disorder detection using MFCC, fundamental frequency and spectral centroid (VDDMFS) emerges as a clear front runner in terms of accuracy, sensitivity, and specificity. VDDMFS achieves an impressive accuracy score, outperforming various ML models commonly applied in the field^62,67^.

Moreover, its sensitivity, standing at 95.36%, ranks as the second-highest among the models considered, reflecting its exceptional ability to identify positive cases. Equally remarkable is its specificity, which registers at 96.49%, which is also the second-highest in comparison. These remarkable metrics underscore VDDMFS’s efficacy in striking a balance between sensitivity and specificity, a critical aspect in voice disorder diagnosis. The model’s ability to maintain such high levels of accuracy while excelling in both sensitivity and specificity positions it as a potent tool in the realm of voice disorder detection. In summary, a comprehensive evaluation of voice disorder detection using MFCC, fundamental frequency and spectral centroid (VDDMFS) was presented. The performance metrics unveiled an exceptional diagnostic process, with an accuracy of 95.67%, marking VDDMFS as a frontrunner in voice disorder detection. Moreover, the model showed a sensitivity of 95.36%, specificity of 96.49% and f1 score of 96.9%, signifying its proficiency in identifying positive and negative cases, respectively.

The proposed approach had an initial memory usage of 532.4 MiB, gradually increasing as pre-trained machine learning models are loaded, physiological signal data undergoes processing, and various feature extraction steps unfold. The peak memory usage reaches 713.6 MiB upon completion. Simultaneously, the application demonstrates a time profile with CPU times revealing user and system components totaling 381 ms, and a wall time of 14 s, encapsulating the overall elapsed time. Understanding these metrics is critical for optimizing the application's efficiency, especially in scenarios involving extensive datasets or resource-constrained environments. For optimal performance, it is recommended to have approximately 800 MiB of free memory and attention to potential runtime considerations.

The combination of Mel-frequency cepstral coefficients (MFCC), fundamental frequency (F0), and spectral centroid has proven effective in identifying voice disorders due to their ability to capture complementary aspects of vocal quality. MFCCs capture spectral features that can reveal changes in vocal tract configuration^56,68^, while F0 reflects pitch variations related to vocal fold irregularities^69,70^. Spectral centroid, indicating spectral energy distribution, aids in detecting anomalies in voice production^71,72^. This hybrid approach provides a comprehensive view of vocal characteristics, enhancing the accuracy of disorder identification.

## Discussion

The proposed model for voice disorder detection exhibits robust strengths that underscore its effectiveness in clinical applications. A key advantage lies in its comprehensive feature set, incorporating Mel-frequency cepstral coefficients (MFCCs), fundamental frequency, and spectral centroid. This inclusive approach ensures a thorough characterization of both spectral and temporal aspects of voice signals, enhancing the model's ability to discern nuanced patterns associated with various voice disorders. The utilization of the latest dataset further fortifies the model's relevance and alignment with current trends in voice disorders, contributing to its real-world applicability. Transparent preprocessing steps, notably the detailed explanation of silent interval trimming, enhance the quality and relevance of the input data. The ensemble approach, amalgamating various machine learning models, contributes to an enhanced overall predictive performance. The model's consideration of both precision and recall, exemplified by the F1 score, ensures a balanced evaluation, particularly crucial in medical applications where misclassification consequences can be significant. These strengths collectively position the proposed model as a reliable and robust tool for voice disorder detection in healthcare settings.

However, it is imperative to acknowledge certain limitations that warrant consideration. The primary constraint lies in the relatively modest dataset size, potentially affecting the model's generalizability. The limited representation of voice pathologies in the dataset necessitates ongoing efforts to expand and diversify it, addressing issues of bias and ensuring broader applicability. Future research should explore collaborations with additional healthcare institutions to acquire a more extensive and diverse dataset. Additionally, the study's focus on binary voice disorder detection highlights the need for future investigations to encompass comprehensive and multiclass voice disorder classifications. The model's dependence on high-quality recording equipment raises practical concerns, emphasizing the need for robust preprocessing techniques to handle variations in recording quality. Despite these limitations, ongoing and future research directions aim to refine the model's robustness, interpretability, and inclusivity, advancing its effectiveness in diverse clinical scenarios.

## Conclusion and future scope

Voice disorders pose a significant health concern, with their prevalence steadily increasing. Early and accurate detection of these disorders is crucial for timely intervention and treatment. In this context, this study aimed to introduce an innovative approach to voice disorder detection, known as VDDMFS, by leveraging machine learning algorithms and various vocal features. It represents a significant leap forward in the field of voice disorder detection, capitalizing on a novel combination of machine learning techniques, including XGBoost, artificial neural networks, and LSTM. The essence of VDDMFS lies in its unique fusion of multiple vocal features, encompassing Mel-frequency cepstral coefficients, fundamental frequency, and spectral centroid. This hybrid model was meticulously crafted to enhance accuracy levels in voice disorder detection. The novelty lies in the amalgamation of these diverse machine learning algorithms, which yielded remarkably high accuracy, with a notable 95.67% accuracy rate. The VDDMFS approach demonstrated a sensitivity of 95.36%, specificity of 96.49% and f1 score of 96.9% for binary voice disorder detection. Such high f1 score, sensitivity and specificity levels are indicative of the potential of this approach in clinical settings. This research not only addresses the growing prevalence of voice disorders but also demonstrates the potential of machine learning in advancing diagnostic methodologies.

Looking ahead, there are promising avenues for further exploration and advancement in this domain. Future research should focus on clinical implementation, longitudinal studies, multimodal analysis, and personalized voice rehabilitation programs to deepen our understanding of voice disorders and their management. Longitudinal studies can provide insights into the progression of voice disorders, treatment efficacy, and potential preventive measures. Real-time voice analysis using wearable devices or smartphone applications represents an innovative approach for continuous monitoring, providing alerts for potential issues and encouraging early intervention. Validating the proposed approach in diverse populations is crucial to ensure its applicability across a wide range of demographics.

### Data collection methods

The data used for analysis and extraction were voice samples obtained from an existing dataset VOICED database, and were not directly collected by us. As specified in the guidelines of Nature Scientific Reports, no direct collection or experimentation involving human subjects or their tissue samples was conducted by us for this study. No additional ethical approval was required for the present study, as the dataset used was de-identified and openly available for research purposes, adhering to the ethical standards of its original collection (Supplementary Information).

### Supplementary Information

Supplementary Information 1.

Supplementary Information 2.

## Data Availability

All data generated or analysed during this study are included in this published article and its supplementary information files.
